# Expression of the HBZ and tax genes of HTLV-1in infective dermatitis associated with HTLV-I (IDH)

**DOI:** 10.1186/1742-4690-8-S1-A252

**Published:** 2011-06-06

**Authors:** Everton S Batista, Isabela Archanjo, Kiyoshi Fukutani, Maria deFatima P de Oliveira, Achilea C Bittencourt, Lourdes Farre

**Affiliations:** 1 Laboratory of Experimental Pathology, CPQGM, FIOCRUZ, Salvador, Bahia, 40296710, Brazil; 2 Laboratory of Immunoparasitology, CPQGM, FIOCRUZ, Salvador, Bahia, 40296710, Brazil; 3 Department of Internal Medicine, HUPES, Federal University of Bahia, Salvador, Bahia, 40296710, Brazil; 4 Department of Pathology, HUPES, Federal University of Bahia, Salvador, Bahia, 40296710, Brazil

## Background

Recent studies have shown that basic leucine zipper factor (HBZ) gene was transcribed in all adult HTLV-1 infected individuals examined, including ATL and HAM/TSP patients, whereas tax mRNA was only transcribed in a half of these groups. The amount of HBZ mRNA expression per provirus was higher than tax mRNA expression in HAM/TSP and adult asymptomatic carriers. Nevertheless, there are no data on HBZ and tax mRNA expression in child and adolescent carriers. The aim of this study was to quantify the HBZ and tax mRNA expression levels in infective dermatitis associated with HTLV-1 (IDH).

## Materials and methods

RNA was extracted from PBMC of 31 patients with IDH and 8 adolescent asymptomatic carriers. The mRNA expression levels were quantified by real-time quantitative PCR. For HBZ gene, spliced (HBZ-SI) and non-spliced (HBZ) transcripts were analyzed.

## Results

The HBZ mRNA expression levels were higher than the tax mRNA expression levels in patients with IDH and in the asymptomatic carriers, for both HBZ-SI (p<0.0001 and p=0.006, respectively) and HBZ (p=0.0019 and p=0.005) transcripts. The HBZ-SI mRNA load was greater than the expression of the HBZ transcript (p=0.0005) only in IDH patients. HBZ-SI and HBZ mRNA load positively correlated with tax mRNA load (p=0.0014, r=0.548 and p=0.0001, r=0.640; respectively) in IDH patients but not in asymptomatic carriers. There was no correlation between the expression of these genes and proviral load in the two groups evaluated.

## Conclusion

These findings suggest that expression of HBZ gene plays a role in early stages of HTLV-1 infection.

